# BrUOG360: A Phase Ib/II Study of Copanlisib in Combination with Rucaparib in Patients with Metastatic Castration-Resistant Prostate Cancer (mCRPC)

**DOI:** 10.1158/2767-9764.CRC-25-0651

**Published:** 2025-12-10

**Authors:** Andre L. De Souza, Matthew J. Hadfield, Shaolei Lu, Dragan J. Golijanin, Roxanne Wood, Jeannine Margolis, Kate Anderson, Adam J. Olszewski, Sheldon L. Holder, Wafik S. El-Deiry, Rahul R. Aggarwal, Anthony E. Mega, Benedito A. Carneiro

**Affiliations:** 1 Legorreta Cancer Center at Brown University, The Warren Alpert Medical School, Brown University, Providence, Rhode Island.; 2 Division of Hematology/Oncology, Department of Medicine, The Warren Alpert Medical School, Brown University Health, Providence, Rhode Island.; 3 Department of Pathology, Brown University, Providence, Rhode Island.; 4 Department of Urology, Brown University, Providence, Rhode Island.; 5 Brown University Oncology Group, Brown University, Providence, Rhode Island.; 6 Brown University Health Cancer Institute, East Greenwich, Rhode Island.; 7 Helen Diller Family Comprehensive Cancer Center, University of California San Francisco, San Francisco, California.

## Abstract

**Purpose:**

Metastatic castration-resistant prostate cancer with homologous recombination (HR) deficiency (HRD) is sensitive to PARP inhibitors (PARPi). PI3K inhibitors (PI3Ki) sensitize to PARPi by disrupting HR in preclinical models. This phase Ib/II study investigated copanlisib (Copa; pan–class I PI3Ki) and rucaparib (R).

**Patients and Methods:**

Eligible patients had metastatic castration-resistant prostate cancer treated with ≥1 androgen receptor inhibitor and taxanes. Phase I followed a standard 3 + 3 dose-escalation design (rucaparib 400–600 mg orally twice a day; Copa 45–60 mg i.v. on days 1, 8, and 15). Primary goal of phase I was to establish MTD and the recommended phase II dose. The primary endpoint of phase II was PSA50 response rate.

**Results:**

Thirteen patients enrolled had a median age of 64 (55–78) years and PSA was 11.7 ng/mL (0.018–2,101). Seven patients received taxanes, and one patient received PARPi. Eight patients had HRD+ tumors [*BRCA2* (four), *BRCA1* (one), *RAD51C* (one), *CDK12* (one), and *FANCA* (one)] and three had *PTEN* loss. There were two dose-limiting toxicities in dose level 1 (rucaparib 400 mg twice a day; Copa 45 mg on days 1, 8, and 15): grade 3 rash and aspartate aminotransferase/alanine aminotransferase elevation. Treatment-related adverse events ≥ grade 2 included leukopenia (54%), anemia (38%), rash (30%), fatigue (23%), and neutropenia (23%). Six patients were treated at dose level –1 without dose-limiting toxicities, which was established as the recommended phase II dose (rucaparib 400 mg twice a day + Copa 45 mg on day 1/15). Among patients with HRD+, one had confirmed partial response and three had stable disease by RECIST, including one patient treated with prior PARPi, and three patients had PSA50 responses (23%).

**Conclusions:**

Copa/R had an acceptable safety profile with a signal of efficacy supporting future studies of PARPi with PI3Ki.

**Significance:**

Gene alterations in the PI3K and HR pathways are common in prostate cancer. Based on preclinical studies, the inhibition of PI3K disrupts HR, and PARP blockade induces AKT activation, supporting the combination of PI3Ki and PARPi. Herein, we investigated the safety and preliminary efficacy of the PI3Ki copanlisib and the PARPi rucaparib in patients with or without alterations in HR repair pathway genes.

## Introduction

Prostate cancer is the second-leading cause of cancer death in men (1). Despite favorable responses in the metastatic castration-sensitive setting to anti-androgens and chemotherapy, most patients develop metastatic castration-resistant prostate cancer (mCRPC; ref. 2). Approximately 25% of mCRPC cases display genomic alterations in homologous recombination genes. Some of the genes (e.g., *BRCA1/2*, *PALB2*, and *RAD51*) sensitize tumors to PARP inhibitors (PARPi; refs. 3–6). The efficacy of PARPi has been established in mCRPC with homologous recombination deficiency (HRD) as monotherapy and in combination with androgen receptor pathway inhibitors (ARPI; refs. 7–14). The phase III PROfound study demonstrated the activity of single-agent olaparib in mCRPC with HRD following progression on novel hormonal agents leading to the improvement of progression-free survival and overall survival (7). PARPi have also improved the outcomes of patients with mCRPC when combined with ARPIs (8, 12–14). Nevertheless, PARPi efficacy is limited by the development of resistance through several mechanisms (15). Therapeutic strategies for patients with PARPi-resistant prostate cancer represent a growing unmet clinical need, especially considering ongoing clinical trials investigating PARPi in metastatic castration-sensitive prostate cancer and evidence of PARPi activity in tumors without HRD (8, 13, 16). Combinations of PI3K inhibitors (PI3Ki) with PARPi have the potential to delay or overcome PARPi resistance and improve the clinical outcomes of prostate cancer.

PI3K/AKT/mTOR pathway alterations present in >70% of advanced prostate cancer contribute to carcinogenesis and tumor progression. PI3K suppression disrupts homologous recombination DNA repair and sensitizes cancer cell lines to PARP inhibition even in the absence of DNA repair gene mutations (17). Preclinical studies in prostate cancer models showed synergy between PARPi and PI3Ki, leading to significant antitumor effects. Olaparib combined with BKM120 (later copanlisib) inhibited tumor growth in *PTEN-*/*TP53*-deficient prostate cancer cell lines and *PTEN* wild-type/*TP53*-deleted prostate cancer xenografts (17). Studies in endometrial, small cell lung cancer, breast, and ovarian cancer cell lines also showed that targeting the PI3K/AKT pathway sensitizes cells to PARPi (18–20). PI3Ki compromised the nucleoside synthesis and nucleotide pool required for homologous repair and sensitized BRCA1-/TP53-deficient breast cancer cells to PARPi (18). Conversely, PARPi triggers cellular stress response and assembly of the ATM–NEMO–AKT–mTOR signalosome that hyperactivates AKT in cervical and breast cancer cell lines (21). In fact, the combination of olaparib with the ATK inhibitor capivasertib showed clinical benefit in 44.6% of patients with advanced solid tumors in a phase I trial (22). These results supported the hypothesis that dual PI3K and PARP inhibition could improve clinical outcomes of mCRPC. We describe the results of a phase Ib/II trial investigating safety and tolerability of copanlisib (pan–class I PI3Ki) and rucaparib (PARP-1, -2, and -3 inhibitor).

## Patients and Methods

### Study population

Eligible patients had histologically confirmed prostate cancer with progression of disease despite castrate levels of testosterone (<50 ng/dL) following treatment with abiraterone, enzalutamide, and/or apalutamide. Previous treatment with taxanes in the castration-sensitive or castration-resistant setting was permitted. Prior treatment with PARPi, radium-223, or sipuleucel-T was also allowed but not required. Somatic or germline HRD mutations were not required for the phase I portion of the study. For inclusion in phase II, however, the presence of at least one deleterious/pathogenic alteration in the following genes was required: *BRCA1*, *BRCA2*, *ATM*, *BARD1*, *BRIP1*, *CDK12*, *CHEK1*, *FANCL*, *FANCA*, *PALB2*, *PPP2R2A*, *RAD51B*, *RAD51C*, *RAD51D*, and *RAD54L*. Variants of unknown significance in the above genes were not considered for eligibility. Results of genomic profiling performed on tumor specimens, germline DNA, or circulating tumor DNA were acceptable. Study approval was obtained from the ethics committees at participating institutions and regulatory authorities. All patients provided written informed consent, and the study followed the Declaration of Helsinki and Good Clinical Practice guidelines. The study was registered at clinicaltrials.gov under the ID NCT04253262.

### Study design and treatment schedule

This was an investigator-initiated phase Ib/II multicenter, open-label study of copanlisib in combination with rucaparib. The phase I, dose-escalation portion of the study, aimed to establish the MTD and determine the recommended phase II dose (RP2D) of copanlisib in combination with rucaparib. Phase I followed a standard 3 + 3 escalation design. The dose schema was as follow (Supplementary Table S1): rucaparib (continuous oral administration twice daily) 400 mg [dose level (DL) –1 and 1], 500 mg (DL 2), or 600 mg (DL 3 and 4) and intravenous copanlisib [45 mg on days 1 and 15 for a 21-day cycle (DL –1); 45 mg on days 1, 8, and 15 for a 28-day cycle (DL 1, 2, and 3); and 60 mg on days 1, 8, and 15 for a 28-day cycle (DL 4)]. Dose-limiting toxicities (DLT) were evaluated according to the NCI Common Terminology Criteria for Adverse Events (CTCAE) version 5.0 and defined as grade 4 neutropenia persisting for >7 days, febrile neutropenia, grade 4 thrombocytopenia, or grade ≥3 thrombocytopenia with bleeding, as well as other treatment-related and clinically significant grade ≥3 toxicity classified under CTCAE with the following exceptions: (i) nausea, vomiting, or diarrhea in patients who have received optimal treatment with antiemetics or anti-diarrheal and that downgraded to grade 1 within 72 hours and (ii) <2 weeks of treatment delay secondary to a treatment-related adverse event (AE) deemed possibly, probably, or definitely related to either study drug.

### Safety and efficacy endpoints

Clinical and laboratory assessments were conducted on days 1, 2, 8, and 15 of cycle 1 and day 1 of each subsequent cycle. Radiographic tumor response assessment including CT of the chest, abdomen, and pelvis and bone scan were performed every two cycles. Response assessments for visceral, soft tissue, or adenopathy were performed according to RECIST 1.1 criteria. Response assessments for osseous metastatic diseases were based on the PCWG3 criteria. AEs were graded using CTCAE version 5.0.

### Pharmacokinetic assessments

Plasma levels of copanlisib were measured before dose and up to 24 hours after dose of the first treatment cycle. Plasma concentrations were determined using validated LC/MS-MS analysis. The maximum copanlisib concentration and area under the concentration curve from 0 to 24 hours were calculated.

### Statistical considerations

The primary endpoint of the phase Ib portion of the study was the determination of the MTD and DLT of escalating doses of rucaparib and copanlisib.

The primary aim of phase II was to estimate the preliminary efficacy of the combination of copanlisib and rucaparib in patients with mCRPC carrying mutations in DNA repair genes, as measured by confirmed PSA50 response rate (PSA response as defined by a decrease in PSA over 50% that is confirmed by another PSA level at no less than 4-week interval). A phase II sample size of 20 patients provided 80% power and a unidirectional level of significance of 0.1. This design yields a type I error rate of 0.099 and power of 0.81 when a true PSA response rate is 68%, with a null hypothesis of a PSA response rate of 44% based on the TRITON2 Study, which demonstrated a PSA response rate of 44% in men treated with rucaparib monotherapy as second-line therapy (23).

## Results

### Study population and baseline characteristics

Between April 2020 and November 2022, 13 patients were enrolled and treated at two investigational sites in the United States (Brown University and the University of California San Francisco). The baseline characteristics of patients are shown in Table 1. Representativeness of study participants is summarized in the Supplementary Table S2. The median age was 64 years (range, 55–78). The median baseline PSA was 11.7 ng/mL (range, 0.018–2,101.4). The median number of lines of prior therapies was two (1–4). Eight patients (53.8%) received previous cytotoxic chemotherapy [docetaxel (five), cabazitaxel (one), and docetaxel and cabazitaxel (two)]. Ten patients (76.9%) had received abiraterone and three (23%) enzalutamide. Three (23%) patients had treatment with sipuleucel-T and only one with radium-223. Among the 13 patients enrolled in the study, six (46%) had tumors with *PTEN* alterations (three mutations and three copy-number loss) and eight (61%) harbored HRD mutations (six patients in phase I and two patients in phase II): *BRCA2* (*n* = 4), *BRCA1* (*n* = 1), *RAD51C* (*n* = 1), *CDK12* (*n* = 1), and *FANCA* (*n* = 1; Table 2). HRD mutation was only required for inclusion in the phase II part of the study. Previous treatment with PARPi was permitted in both parts of the study. Six patients with tumors harboring HRD mutations enrolled in phase I, but only one had been previously treated with PARPi.

**Table 1.** Baseline patient characteristics.

Baseline characteristic	*N* = 13
Age (years), median (range)	64 (55–78)
Race, *N* (%)	​
White	12 (92)
African American	1 (8)
ECOG performance status, *N* (%)	​
0	6 (46)
1	7 (54)
Baseline PSA (ng/mL), median (range)	11.7 (0.018–2,101)
Baseline alkaline phosphatase (IU/L), median (range)	103 (62–345)
Sites of metastatic disease, *N* (%)	​
Bone	13 (100)
Lymph node	8 (61)
Visceral	1 (8)
Measurable disease by RECIST, *N* (%)	6 (46)
HRD gene alterations, *N* (%)	​
Germline	3 (23)
Somatic	7 (54)
HRD gene alterations, *N* (%)	​
*BRCA2*	4 (30)
*BRCA1*	1 (7.6)
*RAD51c*	1 (7.6)
*CDK12*	1 (7.6)
*FANCA*	1 (7.6)
Number of prior therapies, median (range)	2 (1–4)
Previous treatments, *N* (%)	​
Abiraterone	11 (84)
Enzalutamide	3 (23)
Docetaxel	6 (46)
Cabazitaxel	3 (23)
Sipuleucel-T	3 (23)
Radium-223	1 (7.6)

Abbreviation: ECOG, Eastern Cooperative Oncology Group.

**Table 2.** HRD pathogenic genomic alterations.

Subject ID	Somatic alterations	Variants	Germline alterations	Variants
01	RAD51c	Copy-number loss	None	—
04	BRCA1	p.R71T LOF	None	—
06	BRCA2	Copy-number loss	None	—
07	None^a^	—	BRCA2	c.5576del
08	BRCA2	Copy-number loss	BRCA2	p.G2140fs
10	CDK12	p.Q244* LOF p.S651fs LOF	None	—
12	FANCA	c.1566+2C>A	None	—
13	BRCA2	Copy-number loss	BRCA2	p.V2969fs

Abbreviation: LOF, loss of function.

a No tumor specimen submitted for next-generation sequencing; circulating tumor DNA analysis revealed the presence of BRCA2 reversion mutation (p.C1893fs) upon disease progression. The patient had been treated with olaparib prior to the enrollment in this clinical trial.

Eleven patients were treated in the phase I portion of the study and two patients in the phase II. The study was closed during the early stages of phase II because one sponsor could not guarantee the supply of rucaparib.

### Safety

Overall, 13 patients experienced treatment-emergent AEs. The most common grade 2 or higher treatment-related AEs (TRAE) across all DLs were leukopenia (*n* = 7; 54%), anemia (*n* = 5; 38%), neutropenia (*n* = 3; 23%), rash (*n* = 4; 30%), fatigue (*n* = 3; 23%), and oral mucositis (*n* = 2; 15%; Table 3). The most common grade 2 copanlisib-related AEs were fatigue (*n* = 3; 23%), anemia (*n* = 3; 23%), and neutropenia (*n* = 3; 23%). Grade 3 copanlisib-related AEs included maculopapular rash (*n* = 2; 15%), aspartate aminotransferase (AST)/alanine aminotransferase (ALT) elevation (*n* = 1; 7.6%), and anemia (*n* = 1; 7.6%). One patient (7.6%) experienced grade 2 hyperglycemia, and two patients (15%) experienced grade 2 hypertension attributed to copanlisib. The most common grade 2 rucaparib-related AEs were fatigue (*n* = 3; 23%), anemia (*n* = 3; 23%), neutropenia (*n* = 2; 15%), and thrombocytopenia (*n* = 1; 7.6%). Grade 3 rucaparib-related AEs were anemia (*n* = 2; 15%), maculopapular rash (*n* = 2; 15%), AST elevation (*n* = 1; 7.6%), and leukopenia (*n* = 1; 7.6%).

**Table 3.** TRAEs.

Preferred term	Rucaparib 400 mg twice a day + copanlisib 45 mg (days 1, 8, and 15; DL 1; *n* = 5)		Rucaparib 400 mg twice a day + copanlisib 45 mg (days 1 and 15; DL –1; *n* = 8)		Total *N* = 13	
	Grade 1/2 *n* (%)	Grade 3/4 *n* (%)	Grade 1/2 *n* (%)	Grade 3/4 *n* (%)	Grade 1/2 *n* (%)	Grade 3/4 *n* (%)
Rash	2 (15)	1 (7.6)	0	1 (7.6)	2 (15)	2 (15)
Anemia	1 (7.6)	1 (7.6)	2 (15)	1 (7.6)	3 (23)	2 (15)
Leukopenia	2 (15)	0	0	1 (7.6)	2 (15)	1 (7.6)
Neutropenia	1 (7.6)	0	2 (15)	0	3 (23)	0
Thrombocytopenia	1 (7.6)	0	0	0	1 (7.6)	0
Fatigue	1 (7.6)	0	2 (15)	0	3 (23)	0
Oral mucositis	1 (7.6)	0	1 (7.6)	0	2 (15)	0
AST/ALT elevation	0	1 (7.6)	1 (7.6)	0	1 (7.6)	1 (7.6)
Hypertension	0	0	2 (15)	0	2 (15)	0
Hyperglycemia	0	0	1 (7.6)	0	1 (7.6)	0

Two DLTs occurred among five patients treated in DL 1 (rucaparib 400 mg twice a day orally daily; copanlisib 45 mg i.v. on days 1, 8, and 15 for a 28-day cycle) and included grade 3 maculopapular rash and grade 3 AST/ALT elevation attributed to both drugs. The maculopapular rash associated with fever (104°F) occurred during cycle 1 day 9. It was managed with hydrocortisone cream, antihistamines, and systemic corticosteroids. The patient resumed treatment with dose reduction of copanlisib (30 mg on days 1, 8, and 15) without recurrence of rash during five treatment cycles. Another patient experienced grade 3 AST/ALT elevation, which was attributed to both study drugs, leading to study discontinuation. Further evaluation by hepatology revealed steatosis with advanced liver fibrosis related to chronic non-alcoholic steatohepatitis, possibly contributing to liver toxicity. The elevation of AST/ALT returned to normal levels within 20 days after treatment interruption without additional intervention. Six additional patients were treated at DL –1 (rucaparib 400 mg twice a day orally daily; copanlisib 45 mg i.v. on days 1 and 15 for a 21-day cycle) without DLTs. DL –1 was established as the RP2D. Two additional patients were treated at this DL in the phase II portion of the study without significant AEs.

### Pharmacokinetics

Plasma concentration of copanlisib (BAY 80-6946) was measured in samples from nine patients collected on cycle 1 day 15 before infusion (before dose) and at the following time points after infusion: 0.5, 1, 2, 4, 6, 8, and 24 hours. In this pharmacokinetic (PK) cohort, eight patients received copanlisib 45 mg and one patient received 30 mg. Plasma concentrations were used to calculate PK parameters.

**Figure 1.**
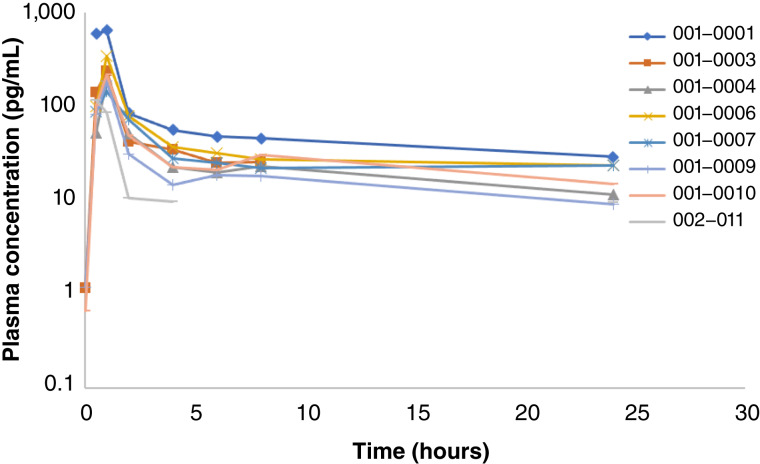
Plasma concentration for copanlisib (BAY 80-6946). Plasma concentration of copanlisib (BAY 80-6946) was measured in samples from nine patients collected on cycle 1 day 15 before infusion (pre-dose) and at 0.5, 1, 2, 4, 6, 8, and 24 hours after infusion. Eight patients received copanlisib 45 mg and one patient received 30 mg. The time to the maximum concentration of copanlisib when administered in combination with rucaparib ranged between 0.5 and 4 hours. The mean terminal half-life was 41.4 ± 56.7 hours. The mean clearance was 32,656 ± 18,164 mL/hour. These parameters are consistent with historical PK parameters of copanlisib.

The time to the maximum concentration of copanlisib when administered in combination with rucaparib ranged between 0.5 and 4 hours. The mean terminal half-life was 41.4 ± 56.7 hours. The mean clearance was 32,656 ± 18,164 mL/hour (Fig. 1). These parameters are consistent with historical PK results of copanlisib [terminal half-life of 38.2 hours (coefficient of variation, 43%)], suggesting that co-administration with rucaparib did not affect the copanlisib profile (24).

### Efficacy

**Figure 2.**
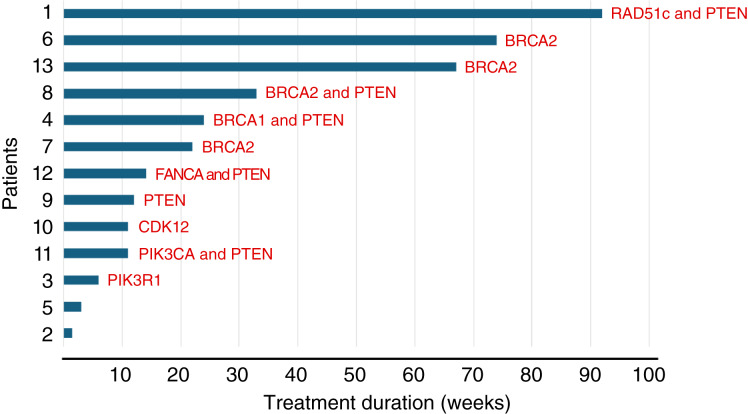
Swimmer plot showing treatment duration on study for all patients. Genomic alterations in the homologous recombination DNA repair and PI3K/AKT/mTOR pathways are noted for each patient.

Ten patients had evaluation of response by PSA. Three patients were not evaluable because of early progression (*n* = 2) and treatment discontinuation due to toxicity (*n* = 1) before the 12-week assessment time point. Three of 10 patients had confirmed PSA50 response (30%). Six patients had measurable metastatic disease per RECIST 1.1 criteria. Among the six patients, one patient achieved confirmed partial response (PR; 16%), and three (50%) had stable disease (SD) by RECIST 1.1 criteria, resulting in a clinical benefit rate of 66%. Among the eight patients with HRD tumors, three had confirmed PSA responses, one confirmed PR, and three SD by RECIST 1.1. One of the three patients with SD had received PARPi (olaparib). The only patient with confirmed PR by RECIST had a tumor with somatic *BRCA2* loss and had not received PARPi. Three patients had alterations in the PI3K pathway, including *PIK3R1* and *PIK3CA* mutations. Six patients had *PTEN* alterations [*PTEN* loss (three) and *PTEN* mutations (three)]. Treatment duration, HRD, and PI3K mutational status are displayed in Fig. 2. At the time of data cutoff (May 8, 2024), nine patients (69%) discontinued treatment because of disease progression, two patients (15.4%) withdrew consent, one patient terminated treatment because of toxicity (elevated AST/ALT), and one patient participating in the phase II came off study because of study closure. This patient with *BRCA2*-mutated prostate cancer had a prolonged PSA response and improvement of bone metastases during 15 cycles (14 months of treatment) with ongoing PSA response when he discontinued his participation in the clinical trial and transitioned to olaparib. Another patient with a tumor harboring *RAD51C* (copy-number loss), *PIK3R1* (c1986-2A>G), *TP53* (p.P278R), *SMAD4* (pC363Y), *PTEN* (copy-number loss), *TMPRSS2-ERG*, and *MYC* copy-number gain had marked improvement of bone metastases, PSA response, and SD per RECIST. Germline testing revealed two variant of unknown significance: *PALB2* (c298C>T p.L100F; missense change without significant impact on protein function) and *PIK3CA* (c483T>C). This patient remained on treatment for 22 cycles (21 months) before developing worsening of bone metastases and progression of prostate tumor with invasion of the bladder. The presence of *RAD51c* loss, *PIK3R1* alteration, and *PTEN* loss possibly contributed to the treatment response. The *PIK3R1* loss-of-function variant—encoding a regulatory subunit of PI3K—together with *PTEN* loss likely resulted in the activation of the PI3K–AKT–mTOR pathway contributing to copanlisib activity. Of note, one patient with a *BRCA2* reversion mutation had SD per RECIST 1.1 and remained on treatment for 5 months. This result suggests the potential of the combination of copanlisib and rucaparib to overcome this resistance mechanism to PARPi.

## Discussion

The study investigated the safety, tolerability, and preliminary efficacy of rucaparib in combination with copanlisib in patients with mCRPC. To our knowledge, this is the first clinical trial to evaluate a PARPi plus a selective class I PI3Ki in mCRPC. The DLTs were grade 3 AST/ALT elevation and grade 3 maculopapular rash attributed to rucaparib and copanlisib. The RP2D was identified following the adjustment of copanlisib schedule from days 1, 8, and 15 to days 1 and 15. The combination at the RP2D showed a favorable safety profile with manageable TRAEs.

The most common grade 2 or higher TRAEs were leukopenia (*n* = 7; 54%), anemia (*n* = 5; 38%), neutropenia (*n* = 3; 23%), rash (*n* = 4; 30%), fatigue (*n* = 3; 23%), and oral mucositis (*n* = 2; 15%). These AEs were consistent with established toxicity profiles for copanlisib and rucaparib. The most common grade 3 TRAEs attributed to copanlisib in previous studies were hyperglycemia, diarrhea, hypertension, and neutropenia (25–27). In a lymphoma study, AST and ALT elevations occurred in 28% and 23%, respectively (28). Another study in marginal zone lymphoma reported similar rates of AST/ALT elevations (31.8% and 27.3%; ref. 29). Both studies reported a 13% incidence of maculopapular rash associated with copanlisib. The most common DLTs related to copanlisib in previous studies were hyperglycemia and hypertension, both occurring in approximately 40% of patients. Neither toxicity emerged as a DLT in our study and did not limit the combination of copanlisib with rucaparib. The AEs observed in the present study were consistent with those associated with rucaparib monotherapy and included fatigue, nausea, vomiting, anemia, and AST/ALT elevation (30–32). Skin toxicities, including maculopapular rash, occur in approximately 27% of patients treated with rucaparib (33). Given that both rash and AST/ALT elevation have been reported with copanlisib and rucaparib, these toxicities were attributed to both drugs in the present clinical trial. The frequency of these AEs in the present study does not suggest a potential exacerbation of AEs with the combination of copanlisib and rucaparib. No new safety signals were observed.

PARPi represent an important component of the standard-of-care treatment of patients with mCRPC harboring homologous recombination DNA repair mutations. They have been approved by the FDA as monotherapy and in combination with ARPIs (e.g., olaparib/abiraterone and enzalutamide/talazoparib) in earlier treatment lines (13, 34–36). As PARPi use expands to earlier disease stages, developing treatment strategies to overcome resistance becomes increasingly important. Several trials are investigating PARPi combinations, including with AKT inhibitors. Olaparib with capivasertib (AKT inhibitor) showed encouraging results in BRCA2-mutated mCRPC and other solid tumors (22). However, PI3Ki might be superior partners for combination with PARPi compared with AKT inhibitors as reduction of the nucleoside pool through glycolytic flux seems to depend on PI3K rather than AKT signaling (37). Combinations targeting other DNA repair pathways such as ATR, USP1, and POLQ with PARPi are also under investigation (38, 39). However, additive myelosuppression can limit these approaches.

This trial is the first to explore copanlisib plus rucaparib in prostate cancer. The regimen was well tolerated at the RP2D, with no new safety concerns or exacerbation of adverse effects. Grade 3 rucaparib-related AEs were anemia (*n* = 2; 15%), maculopapular rash (*n* = 2; 15%), AST elevation (*n* = 1; 7.6%), and leukopenia (*n* = 1; 7.6%). This side effect profile compares favorably with those reported in the TRITON2 and TRITON3 studies of rucaparib in mCRPC (grade 3 rucaparib-related AEs: anemia 24%–25%, rash 0%–1%, AST elevation 5%, and leukopenia 0%–7%; refs. 11, 34). Tumor responses by PSA and RECIST criteria occurred mainly in HRD-positive tumors and were consistent with the results from phase II and phase II studies of rucaparib in mCRPC (TRITON2 and TRITON3). In the rucaparib studies, there was no difference in overall response rate between germline and somatic *BRCA* alterations or *BRCA1* or *BRCA2*. In the TRITON2 study, however, PSA responses were observed more commonly in patients with *BRCA2* alterations and at a higher proportion in patients with biallelic and homozygous *BRCA* alterations compared with the overall population. One of our patients treated previously with PARPi harboring a *BRCA2* reversion mutation (and no *PI3KCA* or *PTEN* alterations) had SD by RECIST 1.1 and remained on treatment for 5 months. This result raised the possibility that copanlisib and rucaparib could overcome the resistance to PARPi provided by the *BRCA2* reversion mutation.

Limitations of this study include the small patient population (*n* = 13) and premature phase II closure due to restricted support and access to rucaparib. The clinical trial is also limited by the lack of correlative studies planned for phase II. Although certain patients had baseline next-generation sequencing in the tumor, longitudinal assessment of circulating tumor DNA and circulating tumor cells was not performed during the clinical trial. Nonetheless, the results support further evaluation of dual PARP/PI3K blockade, particularly in the ∼4% of patients with mCRPC with concurrent HRD and PI3K alterations. Future studies could also investigate combinations of novel PARP1-selective agents with PI3K and/or AKT pathway inhibitors.

In summary, the results of this study support the safety and efficacy of copanlisib in combination with rucaparib at the RP2D in patients with mCRPC. A randomized clinical trial is required to confirm the clinical benefit and refine biomarkers of response.

## Supplementary Material

Supplementary Table S1 Supplementary Table S1. Dose-escalation Schema

Supplementary Table S2 Supplementary Table S2. Representativeness of Study Participants

## Data Availability

The data generated from this study can be requested to the Brown Oncology Research Group at bruog@brown.edu. Data access to anonymized patient-level data and protocol will be granted after review by the Brown Oncology Research Group executive research team.
