# Supplementary material for: BrUOG360: A Phase Ib/II Study of Copanlisib in Combination with Rucaparib in Patients with Metastatic Castration-Resistant Prostate Cancer (mCRPC)
Source: Cancer Res Commun. 2025 Dec 10;5(12):2142–8. doi: 10.1158/2767-9764.CRC-25-0651 (PMC12690636; doi:10.1158/2767-9764.CRC-25-0651)
Supplement: Supplementary Table S1 — Dose-escalation Schema [file crc-25-0651_supplementary_table_s1_suppst1.docx]

**Supplementary Table S1**

| Dose-escalation Schema | | |
| --- | --- | --- |
| Dose Level | Rucaparib  (PO twice daily continuous) | Copanlisib  (IV) |
| -II | 300 mg | 45 mg (days 1 and 15) |
| -I | 400 mg | 45 mg (days 1 and 15) |
| I | 400 mg | 45 mg (days 1, 8, 15) |
| II | 500 mg | 45 mg (days 1, 8, 15) |
| III | 600 mg | 45 mg (days 1, 8, 15) |
| IV | 600 mg | 60 mg (days 1, 8, 15) |
