# Supplementary material for: BrUOG360: A Phase Ib/II Study of Copanlisib in Combination with Rucaparib in Patients with Metastatic Castration-Resistant Prostate Cancer (mCRPC)
Source: Cancer Res Commun. 2025 Dec 10;5(12):2142–8. doi: 10.1158/2767-9764.CRC-25-0651 (PMC12690636; doi:10.1158/2767-9764.CRC-25-0651)
Supplement: Supplementary Table S2 — Representativeness of Study Participants [file crc-25-0651_supplementary_table_s2_suppst2.docx]

**Supplementary Table S2**

| Representativeness of Study Participants | |
| --- | --- |
| Cancer type(s)/subtype(s)/stage(s)/condition | Metastatic castration-resistance prostate cancer (mCRPC) with disease progression following treatment with ≥1 androgen receptor pathway inhibitor and taxanes. |
| Considerations related to: | |
| Sex | All patients included in the trial were male in alignment with the histological diagnosis. |
| Age | Worldwide, the median age of cancer onset of any cancer type (with the exception of skin cancers) is linked to the median age of the underlying population in that country and can vary by as much as a decade. Median age at diagnosis of metastatic prostate cancer is approximately 67 years old and is aligned with the median age of our the study population. ^1, 2, 3^ |
| Race/ethnicity | Worldwide, diagnosis of cancer by race again correlates with the population of a particular country. In the US, cancer incidence per 100,000 is 437 for White; 335 for Hispanic; 427 for Black and 259 for Asian/Pacific Islander. However, there are large disparities in incidence of specific cancer types and deaths based on cancer types in the US. For example, Black men have a 1.7X higher incidence and >2X higher death rate from prostate cancer compared to White. ^4^ Our study included both white and African American patients in proportion commensurate to the small size of the study. |
| Geography | This study enrolled patients in the US and patients were enrolled from the Southwest and Northeast regions. |
| Other considerations | The majority of patients enrolled in part 1 were heavily pre-treated (Table 1) reflecting patients with advanced metastatic prostate cancers participating in phase I trials. Patients received a median of 2 prior systemic treatments including standard treatments with androgen receptor pathway inhibitors, docetaxel, cabazitaxel, Rad 223. The study population included patients with mCRPC harboring mutations in DNA repair mutations. |

1. Sung H, Ferlay J, Siegel RL, Laversanne M, Soerjomataram I, Jemal A, Bray F. Global Cancer Statistics 2020: GLOBOCAN Estimates of Incidence and Mortality Worldwide for 36 Cancers in 185 Countries. CA Cancer J Clin. 2021; 71(3):209-249. doi: 10.3322/caac.21660.

2. Bidoli E, Lamaj E, Angelin T, Forgiarini O, De Santis E, Serraino D. Linearity of Age at Cancer Onset Worldwide: 25-Year Population-Based Cancer Registry Study. Cancers (Basel). 2021;13(21):5589. doi: 10.3390/cancers13215589.

3. https://www.cancer.org/cancer/types/prostate-cancer/about/key-statistics.html

4. AACR Cancer Disparities Progress Report, 2022.
